# Linking the Community Health Fund with Accredited Drug Dispensing Outlets in Tanzania: exploring potentials, pitfalls, and modalities

**DOI:** 10.1186/s40545-022-00507-y

**Published:** 2022-12-29

**Authors:** Angel Dillip, Albino Kalolo, Iddy Mayumana, Melina Rutishauser, Vendelin T. Simon, Brigit Obrist

**Affiliations:** 1 Apotheker Consultancy (T) Limited, Health Access Initiative, Dar es Salaam, Tanzania; 2 Department of Public Health, St Francis University College of Health and Allied Sciences, Ifakara, Tanzania; 3 Kilombero Valley Health and Livelihood Promotion, Ifakara, Tanzania; 4 Social Science Department, University of Basel, Basel, Switzerland; 5 Anthropology Unit, University of Dar es Salaam, Dar es Salaam, Tanzania

**Keywords:** Community Health Fund, Accredited Drug Dispensing Outlet, Micro Health Insurance, Retail outlets, National Health Insurance Fund

## Abstract

**Background:**

In low- and middle-income countries, too, public–private partnerships in health insurance schemes are crucial for improving access to health services. Problems in the public supply chain of medicines often lead to medicine stock-outs which then negatively influence enrolment in and satisfaction with health insurance schemes. To address this challenge, the government of Tanzania embarked on a redesign of the Community Health Fund (CHF) and established a Prime Vendor System (Jazia PVS). Informal and rural population groups, however, rely heavily on another public–private partnership, the Accredited Drug Dispensing Outlets (ADDOs). This study takes up this public demand and explores the potentials, pitfalls, and modalities for linking the improved CHF (iCHF) with ADDOs.

**Methods:**

This was a qualitative exploratory study employing different methods of data collection: in-depth interviews, focus group discussions, and document reviews.

**Results:**

Study participants saw a great potential for linking ADDOs with iCHF, following continuous community complaints about medicine stock-out challenges at public health facilities, a situation that also affects the healthcare staff’s working environment. The Jazia PVS was said to have improved the situation of medicine availability at public health facilities, although not fully measuring up to the challenge. Study participants thought linking ADDOs with the iCHF would not only improve access to medicine but also increase member enrolment in the scheme. The main pitfalls that may threaten this linkage include the high price of medicines at ADDOs that cannot be accommodated within the iCHF payment model and inadequate digital skills relevant for communication between iCHF and ADDOs. Participants recommended linking ADDOs with the iCHF by piloting the connection with a few ADDOs meeting the selected criteria, while applying similar modalities for linking private retail outlets with the National Health Insurance Fund (NHIF).

**Conclusions:**

As the government of Tanzania is moving toward the Single National Health Insurance Fund, there is a great opportunity to link the iCHF with ADDOs, building on established connections between the NHIF and ADDOs and the lessons learnt from the Jazia PVS. This study provides insights into the relevance of expanding public–private partnership in health insurance schemes in low- and middle-income countries.

## Background

In line with the Millennium Development Goals, many low- and middle-income countries have initiated micro health insurance (MHI) schemes [1]. The common goal of MHI is to target people working in the informal sector who are excluded from or not served by other health insurances. In Sub-Saharan Africa [2], too, MHI schemes have been introduced to strengthen social health protection and reduce health-related costs incurred by out-of-pocket health expenditure among people who are not formally employed and constitute the majority of the population.

In 2001, Tanzania introduced a voluntary Community Health Fund (CHF) targeting people working in the informal sector to complement the National Health Insurance Fund (NHIF) which became mandatory for all civil servants in the same year [3–5]. Like other MHI schemes, the Tanzanian CHF has repeatedly been found to attain enrolment rates which rarely exceed 10% [6, 7]. Reported reasons for low enrolment include poor participation of potential beneficiaries during the design of the scheme, lack of trust between the involved actors, weak management of the schemes, limited benefit packages, poor quality of care, and especially frequent stock-outs of medicines at health facilities [8–12].

As part of the Health Sector Strategic Plan IV to reach universal health coverage by 2020 under the Sustainable Development Goals, the Tanzanian government thoroughly reassessed the initial CHF approach to transform and upscale it into a national health insurance scheme for the informal sector. The Swiss–Tanzanian Corporation (2020) contributed to this endeavour through re-designing and piloting the CHF in the Dodoma and later in the Shinyanga and Morogoro regions within the framework of the Health Promotion and System Strengthening (HPSS) project from 2010 to 2023.^1^ The HPSS contributed important design features to the “improved CHF” (CHF *Iliyoboreshwa)* that began rolling out nationally in 2018.

Key features of the improved CHF (iCHF) include: a purchaser–provider split; mobile phone enrolment and payment in the communities; an extended benefit package; access to all government health services through a referral system; mixed provider payment (capitation and performance-based); and an IT-based Insurance Management Information System [12, 13].

The HPSS project also supported another initiative to address a key problem of the “old” CHF: the inadequate government supply of medicines in public health facilities through the Medical Stores Department. The Dodoma regional administration and local government developed and tested the Jazia Prime Vendor System (Jazia PVS), using a public–private partnership approach [14]. Jazia PVS enables public health facilities to supplement medicines and supplies that are out of stock at the Medical Stores Department by purchasing them from a single, known vendor per region, guided by transparent and efficient procurement procedures, and including a number of accountability mechanisms [15]. After a successful pilot in the Dodoma region (since 2014) and expanded testing in the Shinyanga and Morogoro regions (since 2016), the government rolled out the Jazia PVS in all regions of Tanzania in 2018 [14].

This paper explores the links between the iCHF and another public–private partnership aimed at increasing access to medicine in Tanzania, the retail drug outlets known as Accredited Drug Dispensing Outlets (ADDOs) or Duka la Dawa Muhimu (“essential drug stores” in Kiswahili). The government first introduced the ADDO programme in 2003 with the aim of improving access to affordable, quality medicine and pharmaceutical services in rural and underserved areas with few or no registered pharmacies [16, 17]. The accreditation of retail drug outlets was based on the standards set out by the Ministry of Health and Social Welfare and the Tanzania Food and Drug Authority, and involved owner and dispenser training, updating the list of essential medicines and criteria for registering the outlets [17]. After a successful test phase, the government began rolling out the ADDO programme across Tanzania in 2006. Up to now, the programme has trained 20,000 dispensers, and the number of ADDOs (14,045) now exceeds the number of public and private health facilities (8000) at all levels of care in the country [18–20]. Several studies have documented the role of ADDOs in improving access to medicine and as a first resort to care in rural as well as urban settings [17, 18]. ADDOs can sell over-the-counter medicines and a limited list of prescription drugs, such as commonly prescribed antibiotics [20]. ADDOs also serve as a platform for a number of public health interventions, such as disease case identification and referral, and in the integrated management of childhood illnesses [17]. The Pharmacy Council of Tanzania regulates the ADDO programme, with dispenser training institutionalized at various zonal training institutes in the country.

The importance of including the private sector in health insurance systems has been recognized as a move toward complementing public sector efforts in improving access to health services [20–23]. In Tanzania, the NHIF incorporated private pharmacies in the year 2000 [24]. It did so to address rural client demand, because 60% of civil servants are teachers who often live in rural areas with no pharmacies [20]. The iCHF, however, continues to focus on the public health system. As Tanzania is on the way toward UHC and combining iCHF, NHIF, and smaller insurance schemes in a Single National Health Insurance [25], this paper argues that it is high time to explore potential ways and prospects of linking ADDOs with the iCHF. This study thus explores potentials, pitfalls, and modalities of linking the iCHF with ADDOs from diverse stakeholders’ points of view.

## Methods

This was a qualitative exploratory study employing different methods of data collection; in-depth interviews, focus group discussions, and document reviews. We conducted interviews on iCHF at different levels of policy making and implementation, private pharmacies and other relevant stakeholders (see Table 1). The study was inspired by the principles of grounded theory [26]. We interpreted data as we collected it in the field and used insights gained in the process to revise the interview guides on an ongoing basis.

**Table 1:** Participants included in the study

		No of informants	Data collection method
National level	MoHCDGEC	1	IDI
	Pharmacy Council	1	IDI
	NHIF	1	IDI
District/Council level	District health officials	19	IDI
	iCHF staff	4	IDI
	Health facility staff	4	IDI
Ward/Village level	Government officials	14	IDI
	iCHF enrolment officers	37	IDI and FGD
	Health facility governing committee	32	FGD
	ADDO owners/dispensers	26	IDI
	Active iCHF members	3	IDI
	Non-active iCHF members	3	IDI
Total		145	

### Study area

Geographically, the study area covers villages in the Morogoro region in South Western Tanzania. In administrative terms, it was carried out in three rural districts and one town council (Kilombero, Ulanga, Malinyi and Ifakara town council). Morogoro is one of the three regions in which the Swiss–Tanzanian Corporation tested the iCHF, while the ADDO programme has been implemented in this region for more than 10 years. To understand the broader view on the prospect of linking ADDOs with the iCHF, the study team also interviewed experts in Dodoma and Dar es Salaam (Tanzania) and Basel (Switzerland). The study was nested within the larger and independent research project “Participation in Social Health Protection” [27], funded by the Swiss National Science Foundation (SNSF) from 2017 to 2021.

### Research team and reflexivity

Although the research team was already familiar with the Swiss–Tanzanian Corporation and especially the Swiss Tropical and Public Health Institute implementing the HPSS project, this qualitative study has grown out of preliminary insights gained during the independent SNSF research project. Field research conducted with the local NGO KV-HELP in villages of the Morogoro region (BO, IM, MR) clearly indicated that the majority of people frequently use ADDOs and wish for a closer collaboration between the iCHF and ADDOs. The team jointly designed this sub-study to explore the pros and cons of such a collaboration more systematically.

Two team members (AD and VS) led the data collection for this sub-study. They recruited and trained four research assistants with experience in conducting qualitative research. All of them were fluent in Kiswahili, the lingua franca spoken in the area, and none of them was involved in the implementation of the iCHF. After each assistant had conducted two pilot interviews, the field team reviewed the data collection process and adapted the interview guides. Supported by local authorities and the KV-HELP team member (IM), they then selected study participants, explained the purpose of the study, sought their informed consent, and conducted the interviews. None of the participants approached refused to take part in the study.

### Study design and sampling

A constructivism paradigm [28] informs this qualitative study: it is based on the assumption that people construct much of what they learn through experience. The study leverages the constructivism perspective to explore the potentials and pitfalls for linking ADDO with the iCHF, based on diverse stakeholders’ experiences and reflections on such experiences. The study uses systematic, non-probability sampling. This type of sampling is not meant to select a random or representative sample from a population. It is purposeful, because it identifies categories of actors at all levels of the administrative structure (i.e., national, regional, district, ward, and village levels), who have different stakes in the study topic and thus allow for an exploration of diverse points of view (see Table 1). Sampling further followed the saturation principle [29], which means that sampling continued until no new information emerged from the responses.

### Data collection procedures

Data collection was carried out from October to December 2019. The main data collection methods were in-depth interviews (IDIs) and focus group discussions (FGDs), using a guide with four-question-sequences (starting with a main question, and then asking questions to follow up on the answer, to probe to clarify and to prompt if necessary)**.** The study team conducted the IDIs at the workplace or home of the participants, the FGDs at district or village offices. The IDIs involved one research assistant/team leader and the respective participant, while two research team members conducted the FGDs with 10 to 12 participants. Most IDIs did not take more than 1 h to complete, the FGDs 1 h and 30 min. All IDIs and FGDs were audio-recorded, but the interviewers also took notes on key issues. All audio-records were transcribed verbatim, and the most relevant passages translated into English. The two authors also carried out a document review of district materials, particularly with regard to trends in iCHF membership from 2016 to 2019.

### Data management and analysis

Recorded interviews were transcribed verbatim by research assistants supporting the team in data collection, and checked for accuracy by research team members (AD, VS and IM). Transcripts were then translated from Kiswahili into English by professional translators and checked for accuracy by the two authors (AD and VS). The first step toward data management and analysis involved team members (AD, VS, and BO) who familiarized themselves with the translated data. Ten transcripts were coded independently by three study team members (AD and two research assistants), taking into account newly emerging topics. The codes were developed using NVivo (Version 12+) computer assisted, qualitative data analysis software. The lists of codes were then reviewed by all three research team members (AD, VS, and BO), before agreeing on the final codes. The two research assistants and two research team members (AD and BO) coded the remaining transcripts. The final codebook was then grouped into themes and approved by all team members (AD, VS, IM, AK and BO). The data analysis was triangulated between different data collection methods, while the interpretation was made in joint discussions among all co-authors.

### Findings

As indicated in Table 1, the study sample covered participants from the national, district, ward, and village levels. Study findings are presented in the four main categories i) medicine availability and the need to link the iCHF with ADDOs, (ii) stakeholders’ views on the potentials of linking ADDOs with the iCHF, iii) stakeholders’ views on the pitfalls of linking ADDOs with the iCHF, and iv) potential modalities of linking ADDOs with the iCHF.

### Medicine availability at health facilities and the need to link iCHF and ADDO

All stakeholder groups acknowledged an improvement in medicine availability at public health facilities. Study participants at the council level associated this improvement with the Jazia PVS that allows health facilities to order medicine from prime vendors. However, they also pointed out that the challenge of medicine shortages still prevails for various reasons.

*“……yes the situation has improved, you cannot compare the situation now and in previous years, but again patients still suffer from medicine stock-outs, taking an example of our facilities, the workload is high, you may order medicine from MSD [Medical Stores Department] which you think may take you a month or more, but within two weeks the medicines may be finished, and when you order medicines, it is not that tomorrow you will receive the same, it may take two weeks, sometimes even three, so in between when patients don’t receive medicines, they complain a lot.”* (District health official, Kilombero)

Responding to probes into the opportunity of prime vendors supporting health facilities during stock-out times, the district official explained;

*“…the process is still the same, a prime vendor is opted when facilities are out of stock, but the challenge here are the processes; for the health facility to order from a prime vendor, MSD has to provide a stock-out note, saying that I don’t have this particular medicine you ordered, sometimes the MSD fails to provide an out-of-stock notification, something that impacts on stock-out timeline as we can’t go to a prime vendor unless it is a big emergency.”* (District health official, Ulanga)

Health facility staff mentioned medicine stock-outs when talking about the size of the population that is served by health facilities. They pointed out that the majority of patients at health centres are children under the age of five, pregnant women, and elderly who are exempt from paying for health care. In one of the health centres in the town council, for example, the health facility responsible reported conducting almost 24 deliveries on a daily basis, including C-sections, services that are exempted by policy but deplete the stock of medicines, and he continued:

*“I don’t get anything here, if those exempted were at least paying for iCHF, there would be something coming to assist the facility, but we end up dispensing almost all medicines to those who are exempted because they are the ones who experience illness frequently, so because of this we are always out of stock”* (Health facility staff, Ifakara town council)

Ward and village level stakeholders, particularly the Health Facility Governing Committee which is responsible for designing and monitoring health developments in their respective localities, shifted the perspective to the experience of those who are ill and visit health facilities. They pointed out that iCHF beneficiaries may receive a diagnosis and prescription, only to be told that medicines are out of stock when they reach the dispensing window:

*“You know people are joining the improved CHF with hopes that they do not need to purchase medicine at drug outlets, but not all medicines that iCHF members are entitled to at health facilities are available; when you reach the dispensing window, the dispenser writes OS, meaning out of stock; out of stock has become a virus that will bring down iCHF efforts”* (Health Facility Governing Committee, Kilombero)

Health facility staff also commented on the users’ experience but claimed that the majority of iCHF beneficiaries were not aware of their insurance coverage, thinking that they could access all services and medicines at any public health facility through iCHF membership. The medical staff, however, had to conform to the national standard treatment guidelines that regulate what services can be provided by whom at which level of the healthcare system. The iCHF staff raised similar points and emphasized that the iCHF was contractually bound to only reimburse the facility for prescriptions that followed the guidelines to ensure quality of care.

Enrolment officers of the iCHF also noted that the situation of medicine availability has improved at health facilities but observed that medicine stock-outs still deterred some people from signing up with the scheme. They often heard people saying, “what does iCHF membership help if you still have to buy medicine out of pocket.” Another common statement was: “Even when you get a diagnosis worth a million shillings, when there are no medicines, the diagnosis is worthless.”

Communities in all study districts expressed their views on the need to link the iCHF with ADDOs to ensure improved access to medicine. Shortage of medicines at health facilities was among the main reasons cited by non-active iCHF beneficiaries for not renewing their membership, and more importantly, the majority preferred to use ADDOs, because they were easily accessible, in geographical as well as social terms. Linking ADDOs with the iCHF was an initiative regarded as vital, not only to complement services at health facilities but also to strengthen the iCHF scheme by improving access to care and medicine for its beneficiaries.

### Potentials for linking ADDOs with the iCHF

The interview partner at the Pharmacy Council, which represents national level stakeholders and oversees the ADDO programme countrywide, considered it as quite obvious that linking the iCHF with ADDOs has great potential:

*“…there is a big potential for linking the iCHF with ADDOs; first, ADDO dispensers are well trained in disease management, moreover, ADDOs have been a platform for a number of health interventions aimed at improving access to care and medicines, a number of ADDOs in the country are already linked with the NHIF, why not with the iCHF?”* (Pharmacy Council, Dodoma)

Study participants from the district level also recognized the potential and were aware of links between ADDOs and the NHIF. CHF coordinators, district medical officers (DMOs), and health facility staff in particular thought linking ADDOs with the iCHF would improve their work environment as this would increase access to medicine, reduce community complaints on shortage of medicines at health facilities, and boost membership enrolment in their respective districts. iCHF district coordinators argued that extending the services of ADDOs to iCHF members would complement government efforts to improve access to medicines.

The town council iCHF coordinator underlined this point by citing an example. Some private health facilities in the town council had been linked with the iCHF, an initiative that was spearheaded by the council health teams. Two private health facilities were reported to have served iCHF beneficiaries for almost a year, and a faith-based referral hospital has now been providing services for almost 3 years:

*“Yes, if we managed to link the iCHF with private health facilities like the hospital of a faith-based organization catering to the whole Morogoro region, then this can also be done with ADDOs. Of course, we had some challenges with private facilities but these could have been solved earlier if each party understands what is in the contract between the two, but I think there is big promise in this; what is needed is to see how this can best be done”* (iCHF staff, Ifakara town council)

At the ADDO level, differences were noted between owners and dispensers. Owners’ thoughts were geared to business and profit, that is, improving business by serving more customers. Dispensers emphasized the recognition of the ADDO cadre as important health providers, something that would improve community trust in the outlets.

Enrolment officers viewed the connection in two ways. First, as an initiative that could bring more members to join the iCHF, aware that access to medicines had been extended to ADDOs; second, as an income generator for themselves, since, for each member enrolled, they retained 10 per cent of the premium.

Both active and inactive iCHF beneficiaries believed that linking the two would improve their access to medicine, knowing that in case medicines were out of stock at public primary health facilities, they could still be accessed through ADDOs. Inactive beneficiaries believed that more members would join the scheme if this option was offered. They pointed out that young, healthy, and busy people often preferred ADDOs to avoid the long queues of mothers and children at primary health facilities. In addition, the ADDOs were modern (*ya kisasa* in Kiswahili), with attractive premises and services run by local entrepreneurs.

### Pitfalls of linking the iCHF with ADDOs

A number of pitfalls in linking the iCHF with ADDOs were also mentioned. At the national level, study participants expressed their concerns that linking the two may overwhelm the newly established iCHF management during the ongoing national rollout. Related to this, and highlighted by study participants, is the fact that HPSS and the government have invested a lot into the Jazia PVS as a way of improving medicine availability at health facilities; it is, therefore, important to first monitor PVS progress before thinking about complementing the public medicine supply chain with an additional public–private partnership. This was also mentioned by council health staff in the study area.

Another potential downside mentioned by both national and district council participants concerns price differences for medicines between the iCHF, which serves public facilities, and ADDOs that operate on a business model. It was said that ADDOs as private, profit-oriented enterprises sold their medicines and other commodities at higher prices and especially for branded products compared to public health facilities under the iCHF:

*“For example, under iCHF the price for a Fragil tablet is 20 Tsh, but the same tablet is sold at ADDOs at a price of 50 Tsh; it is not easy for ADDOs to accept a lower price because they would not make any profit out of it.”* (District health official, Malinyi)

The above view was also supported by ADDO dispensers who gave the example that Amoxicillin capsules are sold at 2000 Tsh for a single dose at health facilities, while the same medicine is purchased at 3000 Tsh at ADDOs. ADDO dispensers maintained that the government should subsidize the price of medicines for ADDOs, or they should be allowed to purchase medicines from the same source as health facilities, something that may help ADDOs to not operate at a loss when attending to iCHF beneficiaries. The last point was supported by district health teams:

*“Perhaps ADDOs should be supported in this, they should be allowed to purchase medicines at selected prime vendors, for example, where the selling price is low, in this way they would be in a position to serve iCHF members.”* (iCHF staff, Ulanga)

Reflecting on the challenges experienced with the affiliation with private health facilities, iCHF coordinators thought that the use of digital technology might be an obstacle hindering successful collaboration between ADDOs and the iCHF. An example was provided for private health facilities that had contracts with the iCHF to provide services to clients. The iCHF scheme uses a digital Insurance Management Information System (IMIS) to manage membership enrolments and renewals, to process claims and provider payments, and other management tasks. When a client visits a health facility, the IMIS helps to distinguish active from non-active iCHF membership. Private health facility staff received training on how to scan iCHF customers before providing treatment. Because they lacked experience with the technology, most of the private health facility staff did not scan the iCHF beneficiaries before treating them, leading to the provision of services to non-active iCHF members and to misunderstandings between the respective facilities and iCHF district offices. Competence in the use of IMIS technology was, therefore, regarded as vital to ensure successful implementation of the link-up.

The limited list of medicines was another reported pitfall. This was emphasized by district pharmacists saying that the list of medicines at ADDOs had not been updated since 2015, leaving the outlets with a narrow range of authorized medicines. With the changing epidemiology of diseases and drug resistance, district pharmacists thought that it was important that the Pharmacy Council kept the list of medicines updated. An example was given referring to NHIF that are linked to pharmacies; pharmacies are said to have an extended list of medicines and may cater for more health conditions than ADDOs. The limited ADDO list of medicines was, therefore, seen as a pitfall preventing improved access to medicines for iCHF beneficiaries.

### Potential modalities for linking ADDOs with the iCHF

Different viewpoints were noted concerning the modality relevant to linking the iCHF with the ADDO scheme. Two modalities mentioned by study participants included (1) district-based and (2) village-based arrangements. The majority of district level participants, also supported by some of their national counterparts, particularly the Pharmacy Council (PC) office, thought the initiative should commence at the iCHF district offices. The iCHF officials would be required to present the matter to the PC, which is in charge of all the ADDOs in the country. Once the idea was accepted by the PC, the iCHF official would again need to present the plan for linking the two to the District Executive Director, who would forward the idea to the DMO. From the DMO, the matter would be presented back to the iCHF offices and the district pharmacist. The iCHF coordinator and the district pharmacist would need to approach ADDO owners, discuss and agree upon guidelines for the provision of services under the iCHF as well as the payment modalities, and sign a contract regarding iCHF service provision at ADDOs.

Another option for a district-based arrangement would be to use the district advisory committee meetings as a platform. They are conducted twice a year under the auspices of the Regional Administrative Secretary’s office; from there the idea would be passed on to the Regional Commissioner and then on to the President's Office Regional Administration and Local Government (PO-RALG) for implementation.

Other district participants were of the view that linking ADDOs with the iCHF should be an initiative that started at the village level and then move up to the district level. It was said that the idea should first be discussed in village committee meetings, then raised to the ward level and then on to the councils, and later be presented to the district commissioner who would have the last say and could propose that the initiative be implemented in his/her district. This could then be concluded with contract arrangements between interested ADDOs and the respective iCHF offices in the districts.

ADDO owners and dispensers supported the modalities presented above and added that outlet owners are among the important stakeholders regarding the initiative of linking the iCHF with ADDOs. This was mentioned, because, in some districts, owners have their own associations and structures, and conduct regular meetings in this capacity. This means that if the initiative is discussed within and blessed by the association, it would be more likely to succeed. For example, if the two are linked, the role of an ADDO owners’ association would be to follow up with iCHF coordinators in case ADDOs who have served iCHF customers are not paid on time. While this applies to the situation in a city council, ADDO owners’ associations in rural districts were reported to be weaker and thus not play a significant role in supporting such a link-up.

Probing more into the setup, study participants at national and council levels pointed out that linking ADDOs with the iCHF should start with a limited number of ADDOs in the districts, namely, such with larger capital resources, an extensive range of medicines, and a solid number of patients on daily basis. This is because ADDO owners are more likely to accept the price of medicine set by the iCHF if they frequently receive patients in their outlets, achieve profit on a daily basis, and can rely on frequent orders for medicines.

*“It is important that we pilot this, I suggest that we first choose ADDOs that have substantial capital because this would mean a continuous provision of services to iCHF beneficiaries, even when reimbursements from iCHF are delayed”* (District CHF coordinator, Ifakara town council)

Some ADDO dispensers proposed that one ADDO in each ward should be linked with the iCHF to attain wider access to medicines. This was also supported by ADDO owners, maintaining that ADDOs have longer opening hours than health facilities, and once a patient has a prescription from a health facility, he/she can access ADDO services at their preferred time.

The study was interested in exploring the modality of payment to ADDOs willing to join the iCHF. District level participants, particularly coordinators, proposed that the process should follow existing procedures as currently done between NHIF and ADDOs. It was said that ADDOs would need to list their monthly expenditure and submit it to an iCHF office. Once the documents are reviewed and found to be correct, the payment is carried out using the provided ADDO account. Among the concerns expressed by district officials to ensure a successful implementation of the plan is to make sure that ADDOs receive payment for services provided to iCHF members on time.

*“It may be a challenge in the beginning, but the important thing is for the ADDO dispenser to receive payment on time, even if it is only a small amount of money; it should be transferred to the ADDO owner’s account on time to ensure that he can run his business smoothly and continue serving clients with no hitches”* (District health official, Malinyi)

Another consideration highlighted by iCHF district officials is the potential use of technology to link data of outlets with the iCHF digital system. This was said to improve transparency, monitor medicine dispensing at outlets, and smooth reimbursement processes.

The study explored envisioned roles for each stakeholder involved in the linking of ADDOs with the iCHF*.* To ensure successful linkage and implementation, study participants reported playing various roles as follows;

- The iCHF district coordinators’ role would involve cross-checking with the ADDO dispensers to confirm whether the medicines dispensed to iCHF members were correct and in accordance with what is written in the prescriptions.
- The district pharmacist role would be to make sure that ADDO dispensers stock recommended medicines and adhere to the premises and treatment standards.
- The ADDO owners and dispensers’ role would be to adhere to what is agreed upon in the contract between them and iCHF authorities and provide services according to what is expected of them.
- An iCHF supervisor was said to have the role of communicating with ADDO dispensers to understand the challenges they faced when attending to patients and trying to solve those challenges through community sensitization activities. They also envisioned communicating with iCHF beneficiaries and trying to understand and solve problems experienced by iCHF customers at the outlets.

All study participants emphasized that in all steps of initiating the link-ups, the following people/authorities should be involved: iCHF offices at district and national levels, district pharmacists, the Tanzania Food and Drug Authority, the Pharmacy Council, regional pharmacists, district medical officers, ADDO owners, ADDO owners’ associations, lawyers, as well as ward and villages authorities. These were said to have the direct and indirect responsibility of ensuring that the link-up was implemented successfully.

## Discussion

This study explored the potential, pitfalls, and modalities of linking the iCHF with ADDOs as a way of improving access to medicine and reducing out-of-pocket expenditure by insurance beneficiaries. Inclusion of the private sector in the CHF was envisioned to complement the public sector’s efforts to improve access to medicine and care among CHF beneficiaries, beginning in 2001 [30–32], but efforts in this direction remained limited. This study was triggered by the public demand for linking the iCHF with ADDOs, since “public primary health facilities prescribe medicines, ADDOs dispense them”, as a common saying goes.

Study participants acknowledged that the Jazia PVS has contributed toward an improvement in medicine availability at health facilities in the country. This finding is well-supported by [14, 15], indicating an increase in the availability of tracer medicines (from an average of 69% in 2014 to 94% in 2018) at public health facilities in Dodoma, where the Jazia PVS was piloted. However, all stakeholder groups in our study reported that medicine stock-outs are still common in public health facilities. This finding tallies with results of a medicine audit assessment carried out in the same year as this study, showing that only 10 per cent of sampled primary health facilities had ten essential tracer medicines in stock for 12 months in 2019 [33].

While there is clear evidence of medicine stock-outs in 2019, community complaints on medicine shortages at health facilities may also reflect unrealistic expectations concerning iCHF benefit packages. This point was raised by health facility staff and may be partially explained by iCHF communication strategies. In sensitization meetings and even on the official website, people are told that CHF members “will have access to all medical services in accordance with the referral procedure at all levels of government service centers” (http://www.chf-iliyoboreshwa.or.tz/en/chf-benefit-package). However, without a detailed explanation of the referral procedure, this statement can easily lead to inflated expectations.

All stakeholder groups saw great potential in linking the iCHF with ADDOs. The Pharmacy Council viewed ADDOs as a useful platform to improve access to medicine due to its wider coverage in the country, skilled dispensers, and the fact that some ADDOs are already linked with the NHIF. The incorporation of ADDOs as private drug outlets into the NHIF is well-elaborated by [20]. Already in 2000, stock-outs of medicines at public health facilities led to the inclusion of ADDOs into the NHIF, and since then, a number of ADDOs have been serving NHIF clients across the country, thus complementing public health service provision.

The professional group (i-CHF coordinators, DMO, and health facility staff) thought that joining the two would improve their work environment, as community complaints regarding medicine stock-outs at health facilities would decrease, and more members would join the scheme, resulting in an increased enrolment and a better functioning of the iCHF. As observed during the interviews, aspiration and personal commitment are key to ensure the success of initiatives. The town council iCHF coordinator was aware of the links between the NHIF and pharmacies/ADDOs and tried to spearhead similar initiatives by linking the iCHF with private health facilities in her council to increase service coverage. ADDO owners, dispensers, enrolment officers as well as active and inactive iCHF beneficiaries expressed the potential of linking the iCHF with ADDOs for the sake of improving access to medicine and complementary services at public health facilities. iCHF enrolment officers in particular expressed how the affiliation could financially benefit them but also increase community enrolment in the scheme.

While acknowledging the importance of linking the iCHF with ADDOs, study participants also highlighted some of the pitfalls of such an arrangement. Leaders at the national level pointed out the recent investments made in establishing the Jazia PVS. Piloting and scaling up the prime vendor model presented many organizational challenges, both to implementers and to the health workforce [14]. This is of course also true of the iCHF. To integrate the ADDO programme would require an additional, concerted effort, but it could build on the experience of the Jazia PVS initiative and the linking of the NHIF and ADDOs.

A second pitfall identified in this study refers to the different pricing mechanisms for medicines between the iCHF and ADDOs. Unlike the NHIF, the iCHF uses a capitation rather than a fee-for-service reimbursement model. Moreover, ADDOs charge higher prices than public health facilities, because they are operating on a profit basis. The price difference was also mentioned as a barrier for a successful affiliation of ADDOs and the NHIF, since ADDO owners considered NHIF prices being below the market price and feared ending up dispensing medicines on a loss basis [20]. Related to this is the limited list of medicines ADDOs are allowed to sell. According to district pharmacists, this list has not been updated since 2015 and no longer complies with the standard treatment guidelines. ADDOs can thus only supplement a limited range of medicines if they are out of stock at primary health facilities [20], but even so they provide a valuable back-up service [12, 34].

A third pitfall pointed out by study participants were inadequate digital skills. The iCHF uses the IMIS as a special digital health solution to facilitate transactions, linking customers through the iCHF administration with public health care facilities [35]. From a technological point of view, private health care providers including ADDOs can be easily integrated into the IMIS, but if they are not sufficiently trained and supervised, this may cause practical problems, as past experiences of iCHF coordinators have shown. As discussed by Obrist et al. (in progress), aspirations toward the use of digital tools may not translate into practice. Private health facilities linked to the iCHF in one of the study districts did not, although trained, use scanners to identify active beneficiaries before providing service, something that contributed to misunderstandings and breaches of contract between the two parties. Moreover, although not mentioned by study participants, the use of electronic health records (i.e., personal data) for purchasing-related tasks, such as claims management and other analytics, raises questions as to data security and data protection based on legislation and informed consent [36].

Further pitfalls not mentioned in our study are fraud and medicine safety. As discussed in the study by [20], health facility staff may pretend that a medicine is out of stock and refer patients to drug retail outlets they own or collaborate with, or dispensers in health facilities or ADDOs may make errors when issuing prescriptions.

Village and district-based modalities were mentioned as a potential to ensure that the link-up of iCHF and ADDOs happens. Leveraging the experiences of linkage between the iCHF and private health facilities, NHIF and private retail outlets, and particularly the use of contractual agreements, all study stakeholders acknowledged the necessity of linking iCHF with ADDOs. Private health facilities providing services to iCHF clients were reported to have signed binding agreements between the two parties. Among other things, the agreements spell out the various services iCHF beneficiaries are entitled to at the respective health facilities, but also the steps that health facilities need to take before offering services to clients. Learning from the contractual agreements between ADDO and the NHIF, national standard treatment guidelines are part of the contractual agreement, ensuring proper payment modalities between the two parties [20]. Similar approaches are emphasized by [37], highlighting the relevance of service agreements between public and private providers as a way of complementing and improving access to health services.

Based on stakeholders’ views and ongoing medicine stock-out challenges at health facilities, this study sees high potential for joining iCHF and drug outlets to improve the availability of medicine among scheme beneficiaries and increase iCHF coverage. Study participants at all levels, enrolment officers, active and inactive iCHF beneficiaries all acknowledged the relevance of ADDOs in increasing access to medicine, envisioning more enrolment if the scheme is linked with the drug outlets. ADDOs have been recognized for their value in improving access to essential medicine, particularly in underserved areas [17, 38], where the majority of CHF beneficiaries live. The iCHF has great potential to improve coverage by incorporating ADDOs in service provision [20].

The feasible modality for linking up the iCHF and ADDO as suggested by a majority of study participants is for the process to start at district/council offices. Responsible iCHF officials need to define criteria for ADDOs interested in joining the scheme and present the plan to the Pharmacy Council before involving other authorities at district level to authorize the plan. Learning from the experiences of linking up retail outlets and NHIF [20], there is need to form an iCHF accreditation committee at district level to defines requirements for ADDOs interested in joining the insurance scheme. There should also be contracts and agreements between iCHF district offices and ADDOs that define, among other things, guidelines for services provision and reimbursement modalities, and pilot the initiative with a few ADDOs, generate learning experiences and assess the feasibility of the approach.

Some of the key pitfalls when joining the two schemes, particularly the price of medicines at ADDOs, could be mitigated by leveraging the experience of retail outlets and NHIF, respective district officials set medicine reimbursement per market survey, and an assessment of other relevant criteria [20] to allow ADDOs to operate on a profit basis. Such an initiative requires a proactive approach and commitment, acknowledging that a similar modality of linking the iCHF with private health facilities in one of the study districts was initiated by the respective district officials. The Tanzania Medicine and Medical Device Authority (TMDA), which oversees ADDO operations in the country, does not have a scheduled plan to update a list of medicines at ADDOs, bring together TMDA, iCHF authorities, and the Pharmacy Council, and to establish a plan around regular updates of lists of medicines at retail outlets that could improve access to medicines for insurance beneficiaries [20].

The Tanzania digital health strategy 2019–2024 [39] embraces the application of digital health technologies to improve the quality of care and management of human and financial resources. A successful linking of the iCHF with the ADDO scheme would require the use of digital technologies and the Insurance Management Information System (IMIS) to improve communication between the two parties, track health facility medicine stocks, and prescription practices [20].

## Conclusions

The iCHF has never been able to fully resolve the problem of low engagement owing to frequent medicine stock outs at health facilities. While the Jazia PVS initiative has improved the situation of medicine availability at health facilities, stock-outs are still common. As the government of Tanzania is moving toward the Single National Health Insurance Fund, there is a great opportunity to link the iCHF with ADDOs to ensure broad access to medicine, considering the widespread presence of ADDOs in the country. District health teams, particularly iCHF coordinators, have a crucial role to initiate the link-up by piloting an approach with a few selected ADDOs. The Jazia PVS and NHIF/private retail outlet linkage provides a good and informative model of joining ADDOs with the iCHF. These findings are of more general relevance for expanding public–private partnerships in health insurance schemes in low- and middle-income countries.

## Data Availability

Copies of the interview guides, transcripts, and audio data are available from the first author.

## Abbreviations

ADDO: Accredited Drug Dispensing Outlets
CHF: Community Health Fund
iCHF: Improved Community Health Fund
MHI: Micro Health Insurance
NHIF: National Health Insurance Fund
PVS: Prime Vendor System

## Footnotes

1 The HPSS project was funded by the Swiss Agency for Development and Cooperation and implemented by the Swiss Tropical and Public Health Institute.
